# On the evolutionary language game in structured and adaptive populations

**DOI:** 10.1371/journal.pone.0273608

**Published:** 2022-08-30

**Authors:** Kaloyan Danovski, Markus Brede

**Affiliations:** Electronics and Computer Science, University of Southampton, Southampton, Hampshire, United Kingdom; Kyushu Daigaku, JAPAN

## Abstract

We propose an evolutionary model for the emergence of shared linguistic convention in a population of agents whose social structure is modelled by complex networks. Through agent-based simulations, we show a process of convergence towards a common language, and explore how the topology of the underlying networks affects its dynamics. We find that small-world effects act to speed up convergence, but observe no effect of topology on the communicative efficiency of common languages. We further explore differences in agent learning, discriminating between scenarios in which new agents learn from their parents (vertical transmission) versus scenarios in which they learn from their neighbors (oblique transmission), finding that vertical transmission results in faster convergence and generally higher communicability. Optimal languages can be formed when parental learning is dominant, but a small amount of neighbor learning is included. As a last point, we illustrate an exclusion effect leading to core-periphery networks in an adaptive networks setting when agents attempt to reconnect towards better communicators in the population.

## Introduction

The central role played by human language in the evolution of our species has been widely accepted, so it is unsurprising that its emergence and evolution has received attention from various fields, from linguistics to neuroscience and evolutionary biology [1]. A central question in the emergence of language is how biological, social, and learning factors combine into complex dynamics that allow for systems of communication to emerge [2, 3]. However, it is notoriously difficult to make any quantitative claims on this subject due to the lack of empirical evidence from the time periods of interest [4].

One tool employed in solving this issue are computational simulations, which focus on the development and testing of formal models of language and communication, based on linguistic hypotheses. The aim of such studies is to understand the processes that result in the emergence and stability of communication systems without a central authority imposing universal rules of behavior. There is an abundant interdisciplinary literature on the subject of modelling and analyzing language dynamics through the lens of complexity science, with many models inspired by physics, economics, and sociology [5].

All computational modelling of language evolution necessarily relies on some assumptions on the formal representation of language, and simplifications that make it feasible to analyze or simulate its dynamics. Due to the complexity of the system under examination, studies usually focus on isolated features of a language (such as lexicon, syntax, or morphology), although the importance of cross-model comparisons has also been emphasized [6]. Models in this domain formalize a population of individuals endowed with a language and the capacity to learn and interact, resulting in population-level dynamics that are usually complex and non-linear in nature, despite the limited nature of the language representation. In the field of *semiotic dynamics*, the fundamental assumption adopted is that of language as a system of shared conventions, which are represented as mappings between objects (in the world) and words/meanings (that can be communicated). This line of thinking can be traced back to the philosophical studies of Lewis [7] and Wittgenstein’s language games [8]. Objects and words are often fixed, with the focus of study centered on the change in the mappings between them. This approach to studying language evolution echoes principles from the study of the emergence of consensus [9], and hence uses similar methods from evolutionary game theory [10] and statistical physics [11] to analyze the system’s emerging behavior.

One of the first models for the evolution of language as object-signal mappings is the Evolutionary Language Game (ELG) model proposed by Nowak et al. [12]. In the ELG, a population of agents with individual languages and learning strategies is subject to reproduction dynamics based on their ability to communicate with others, resulting in the convergence towards an equilibrium state, where a common language is shared between all agents. Another seminal model, which relies on self-organization rather than evolutionary dynamics, is the Naming Game (NG) model [13], originally proposed by Steels [14]. In the minimal formulation of the NG [15], agents perform pairwise interactions in order to agree on the use of a single word to name an object. The dynamics of the model can result in the emergence of a common word shared between all agents. The NG has been studied thoroughly, see Refs. [16] for an overview. Although the language representation of the minimal NG can be considered as a simplified version of the one used in the ELG [5], the models’ dynamics are qualitatively different. Specifically, the NG proceeds on much shorter time scales [17], thus describing a process of inter-generational (horizontal) coordination, in contrast to the cross-generational (vertical or oblique) transmission of the ELG.

One aspect of NG dynamics that has been studied thoroughly is the effect of the topology of the population’s social network. It has been shown, for example, that small-world effects speed up convergence to a common word while limiting the cognitive demand on agents [18, 19]. Furthermore, the co-evolution of state and topology [20] has also been studied for the NG, most notably by Lipowska and Lipowski [21], who examined the model’s dynamics on a weighted network where agents were more likely to communicate if they had done so successfully in the past. They showed that this model can lead to the same stable, multi-language states that can be observed on static networks with community structure. Although not a strictly adaptive model, since information from the state of nodes does not feed back into the network topology, Maity et al. [22] studied the behavior of the NG on time-varying real-world networks, showing very strong effects of a temporal changing network topology on the convergence dynamics of the model compared to static networks, and posit this has to do with the introduction of new agents into the population. Fu and Zhang [23] also showed that adaptively rewiring links in a biased, two-word NG can significantly affect the speed of convergence. In exploring adaptive network dynamics, we can draw comparisons to the vast literature on co-evolutionary spatial Prisoner’s Dilemma (SPD) models from evolutionary game theory. For example, studies have shown that adaptive rules can promote cooperation ([24, 25], and many others) and that the time-scale of adaptivity of the network compared to that of node states can significantly affect the model dynamics [26, 27]. A comprehensive review is outside the scope of the current work, but these can be found in Refs. [20, 28], and the introduction to [29].

In contrast, neither the effects of static nor adaptive topologies have been explored for the dynamics of the ELG. Extensions of the model have looked at the effects of linguistic and cognitive factors, such as noise in information transmission [30] and learning biases [31], but very few have examined the effect of social structure. An exception to this is the work by Di Chio and Di Chio [32], who studied the effect of spatially embedding the population onto a 2D lattice where agents are allowed to relocate. As a result, a dynamics resulting in language clustering was observed. However, to the best of our knowledge, the general effects of the topology of the population’s social structure, as well as those of adaptive rewiring, have not been explored yet for the ELG model.

The present study aims to examine the effects of social structure on the dynamics of an evolutionary model inspired by the ELG. We look at different kinds of simple and complex networks, in an attempt to draw conclusions about the effects of different network properties (such as degree heterogeneity and small-world effects) on the convergence towards a shared language. We also explore the differences in behavior of vertical and oblique language transmission on different topologies. Furthermore, we model the co-evolution of language and social structure by exploring the model’s dynamics on networks with adaptive rewiring rules.

In the next section, we detail the model, including the types of networks used and the adaptive rules. We then present the results of our simulations using this model, and discuss the effects of topological properties and rules/parameters. We conclude by discussing our findings in a wider context, and give pointers for avenues of further study.

## Model

In this section, we outline the details of a model of a networked population of agents evolving semiotic conventions through a dynamics inspired by the ELG [12]. As a baseline, the model consists of a population of *N* agents, embedded onto a network such that each agent occupies a single node. We consider a setting comprising *n* *objects* and *m* possible *signals* to reference them, and assume that each agent is endowed with their own *language* *L*. We understand a language as an association between objects and signals, and use it to assess the ability to convey information between agents. Language change is modeled via the reproduction of agents and learning (through observation), as new agents replace old agents on the network.

### Language representation and payoff

A language *L* consists of probabilistic mappings between the set of objects and the set of signals, as can be formally defined by two matrices. The *n* × *m active matrix P*, whose entries *p*_*ij*_ represent the probability of producing signal *j* to refer to object *i*; and the *m* × *n passive matrix Q*, whose entries *q*_*ji*_ represent the probability of inferring object *i* from signal *j*. In other words, the rows of the *P* and *Q* matrices represent discrete probability distributions for translating either objects into signals, or signals into objects, respectively.

Consider agents *I*_1_ and *I*_2_, with languages *L*_1_ and *L*_2_, respectively. Hypothetically, communication proceeds as follows. Agent *I*_1_ produces signal *j* in reference to object *i* with probability *p*_*ij*_, as defined in *L*_1_. Agent *I*_2_ tries to infer which object *I*_1_ is referring to, and infers $\hat{i}$ with probability $q_{j\hat{i}}$, as defined in *L*_2_. With probability $p_{ij}^{\left(1\right)}q_{ji}^{\left(2\right)}$, $\hat{i}=i$, and communication is successful.

In practice, we do not simulate the communication process, but consider the *probability of successful communication* as a proxy for agents’ fitness. For agents *I*_1_ and *I*_2_, this probability is the sum $\sum_{i}^{n}\sum_{j}^{m}p_{ij}^{\left(1\right)}q_{ji}^{\left(2\right)}$ over all *n* objects and *m* signals. The symmetric, individual *payoff of communication F* between agents *I*_1_ and *I*_2_ is defined as:

$$\begin{array}{c} F\left(L_{1},L_{2}\right)=\frac{1}{2}\sum_{i}^{n}\sum_{j}^{m}p_{ij}^{\left(1\right)}q_{ji}^{\left(2\right)}+p_{ij}^{\left(2\right)}q_{ji}^{\left(1\right)}. \end{array}$$ (1)

The *total payoff* of agent *I* is its average payoff of communication *F* with all of its neighbors, defined as:

$$\begin{array}{c} F_{I}=\sum_{J}^{M_{I}}\frac{F(L_{I},L_{J})}{|M_{I}|}, \end{array}$$ (2)

where *M*_*I*_ is the set of *I*’s neighbors—all agents that are within one link’s distance from *I* on the social network. This formulation allows us to model frequency-dependent bias towards popular languages, since an agent will have a better chance to propagate his language if the latter is more common within the agent’s neighbourhood. In this way, we account for a scale-merit or bandwagon effect in the propagation of languages, which is a significant factor affecting linguistic development in the real world. See S1 Table for further discussion and results illustrating this point. However, we normalize by the number of neighbors |*M*_*I*_| in order to avoid bias towards popular agents from linguistic factors (see Ref. [33] for an example of this effect). We are still interested in bias towards popular agents that emerges from the topological properties of the social network, and their place within it. For example, better connected agents have more influence on individual- and population-level payoffs.

#### Reproduction and learning

We adopt an evolutionary perspective, whereby the state (languages) of the population changes as a result of natural selection on the local communicative success of languages. The total payoff *F* is therefore a proxy for an agent’s evolutionary fitness.

At every time step of the simulation, a *reproduction step* can take place, proceeding as follows. First, a parent is chosen from the entire population with a fitness-proportional probability. Secondly, a child is created and its language sampled from the population. Lastly, the child replaces one of the parent’s neighbors with uniform probability. This update strategy is known as *birth-death* [34], similar to the Moran process in replicator dynamics. A number of other strategies are discussed in Ref. [35]. We explore birth-death updating due to its simplicity and prevalence in other studies of evolution on structured populations [36–40]. While the choice of update strategy can significantly impact the model’s dynamics [19, 34, 41, 42], especially on heterogeneous networks [43], exploring this element has been left for future work.

When a new child is ‘born’, it does not inherit its parent’s language directly. Instead, it learns a new language through observation and imitation. Specifically, it constructs an *n* × *m association matrix A* by sampling responses from its parent and *K* of its parent’s neighbors. Neighbors are chosen with replacement, with a probability proportional to their total payoff *F*. For each of the *n* objects, a child samples *k* signals from each agent in the sample pool (as defined by the sampled agent’s active matrix *P*). The sampled responses from the parent and the neighbors are aggregated and normalized to form two *n* × *m sample matrices*, *S*_*p*_ and *S*_*n*_, respectively, containing the observed discrete probability distributions for each object.

A weighted average of the sample matrices is used to construct *A*. To distinguish the effects of parent-based an neighbor-based learning, we introduce a *neighbor influence* parameter *δ* ∈ [0, 1], which determines the weight of the neighbor samples *S*_*n*_ in the child’s final language. For *δ* = 0, the child only uses the samples from its parent to construct *A*. Conversely, for *δ* = 1 only the neighbors’ sample distributions are used, whereas for *δ* = 0.5 both have equal weight. Thus *A* is constructed as *A* = (1 − *δ*) ⋅ *S*_*p*_ + *δ* ⋅ *S*_*n*_. Finally, the child’s language *L* consists of the *P* and *Q* matrices, which are obtained by normalizing the rows and columns of *A*, respectively, such that:

$$\begin{array}{c} p_{ij}=a_{ij}/(\sum_{l}^{m}a_{il}),\;q_{ji}=a_{ij}/(\sum_{l}^{n}a_{lj}). \end{array}$$ (3)

**Fig 1:**
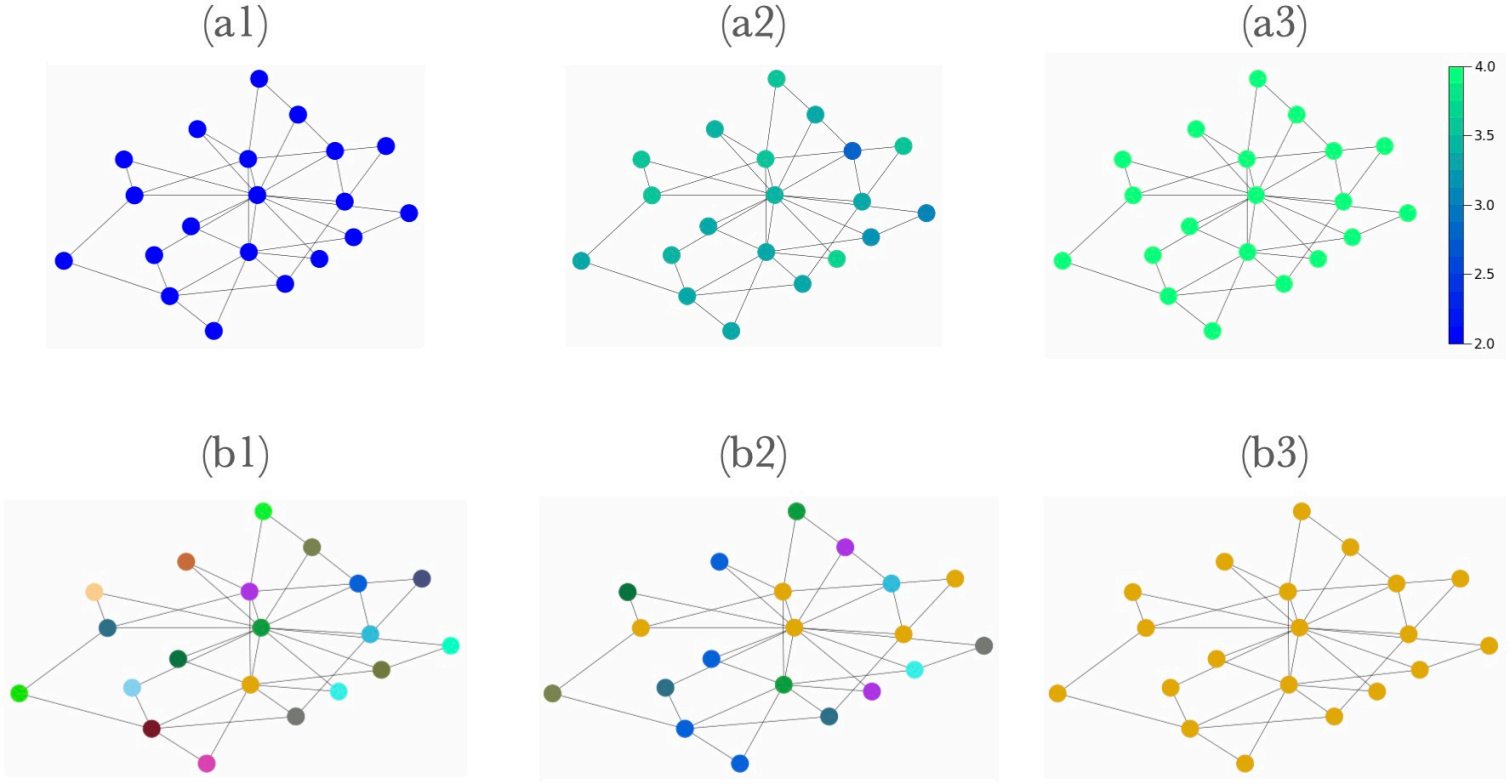
Illustration of the convergence dynamics towards a common language. Each node represents a single agent, and is colored (a) based on the agent’s payoff, with a lighter color implying higher payoff, and (b) based on agents’ languages, with each color representing a distinct language. In the initial generation, all agents are assigned different, randomly generated languages (b1) that are not well-suited for collective communication (a1). Correspondingly, payoffs are similar and low. As the simulation progresses, some languages are adopted by multiple agents (b2), and all languages become more alike, yielding higher payoffs (a2). By the end, all agents adopt the same language (b3), and the payoff of communication is the maximum possible given that language (a3). (Colors between (a) and (b) are not related).

Since the number of samples per object *k* is a finite number, *A* is an imperfect reproduction of the *P* matrices of the sample pool. Over time, this learning process implies that children’s *P* and *Q* will tend towards binary permutation matrices. For *δ* = 0 (purely parental learning) or the case where the parent and all *K* neighbors have the same language, this represents an equilibrium state, or convergence to a common language. This process is illustrated in example simulations in Fig 1. The languages of agents in the first generation are initialized by generating random, non-binary *A*, whose entries are uniformly sampled integers in the range [1, 9].

#### Social structure

The social structure of the population is modelled using simple or complex networks, namely 2D regular lattices, ring (and small-world) graphs, random networks, and scale-free networks. *Lattices* were explored for their regular structure and usefulness for modelling real-world spatial topologies. All lattices explored are square regular grids with a toroidal topology. When discussing “even-sized” and “odd-sized” lattices below, we are referring to the length of their dimensions, e.g. a lattice defined by 36 nodes on a 6 × 6 grid is even-sized. *Ring graphs* were explored because of their longer average path length and higher clustering compared to the rest of the networks studied here. *Small-world* (Watts-Strogatz model [44]), *random* (Erdős–Rényi model [45]), and *scale-free* (Barabási–Albert model [46]) networks were used to explore the effects of degree heterogeneity and short average path lengths, which are common in real-world social networks [47, 48].

#### Adaptive network rules

In some of the results presented below, we also model the co-evolution of the state and topology of networks by introducing the possibility of rewiring events. In order to control the time-scale of language evolution relative to the learning dynamics, we introduce a parameter λ which modes the probability that a rewire event will occur, and let reproduction events occur with probability 1 − λ. The *rewire event* proceeds as follows:

1. An agent *I* is picked at random to rewire.
2. An agent *J*_*old*_ is picked to be disconnected from, based on *I*’s existing links and a disconnection rule.
3. An agent *J*_*new*_ is picked to be reconnected to, from the population, based on a reconnection rule.
4. The edge (*I*, *J*_*old*_) is removed and the edge (*I*, *J*_*new*_) added to the network.

The choice of disconnection rule governs which links will be removed. We have considered three different disconnection rules: (1) *uniform*, in which case a random agent is picked from the set of *I*’s neighbors *M*_*I*_; (2) *fitness-proportional*, in which case the probability of picking agent *J*_*old*_ from *M*_*I*_ is proportional to its total payoff $F_{J_{old}}$; and (3) *fitness-inverse*, in which case the probability of picking *J* is proportional to $1-F_{J_{old}}$. For our purposes, (1) models a base-case, (2) models agents disconnecting from other well-performing agents, which we include as reference, and (3) models real-world scenario with agents attempting to improve payoff by disconnecting from agents that cannot communicate well.

Similarly, the choice of reconnection rule governs which agents in the population will be selected to create a new link. We consider two rules: (1) *uniform*, in which case a random agent is picked from the population *N* (excluding *M*_*I*_); and (2) *fitness-proportional*, in which case an agent *J*_*new*_ is selected from the population (excluding *M*_*I*_) with a probability proportional to its total payoff $F_{J_{new}}$. For the proportional reconnect strategy, we introduce a 10% chance that a random agent will be picked instead, in the same way as with the uniform strategy. This is done to account for noise and to allow isolated agents, with *F* = 0, to be connected to, thus preventing a large number of isolated nodes from accumulating. Again, (1) models a base-case scenario, while (2) models agents taking into account communicative success, as a proxy for social standing, when considering new connections.

In summary, the model we propose is heavily influenced by the ELG, but exhibits a number of important differences. The first is that in our model, an agent’s payoff is determined by their communication efficiency with their neighbors on the social network, rather than with the entire population. The second is that our model does not preserve the discreteness of generations and is not limited to vertical transmission of languages, but rather models a process of oblique cultural transmission that allows us to explore the effects of tuning between parental and neighborhood (role-model) learning.

Below, we employ agent-based simulations to examine the model’s dynamics. In each time step *t* of the simulation, either a reproduction or a rewire step (when considering adaptive networks) occurs, until a preset limit *t*_*max*_ is reached. We impose a time step limit and use comparatively small system sizes, due to constraints on time and computational resources. With all runs parallelized, a configuration with *N* = 400 and *t*_*max*_ = 4 × 10^6^ takes at best 40 hours to run. In general, the runtime of simulations scales linearly with *Nt*_*max*_. Simulations were developed in Python (3.9), using the NetworkX (2.7) and NumPy (1.12) libraries. The code used to run the simulations has been made publicly available (see Ref. [49]).

In the following section, we investigate the effects of different linguistic parameters, network topologies, and rewiring rules on the convergence properties of the simulations.

## Results and discussion

In order to examine the dynamics of the model, we carry out a number of Monte Carlo, agent-based simulations. For all simulations, unless otherwise stated, we use the following setup. There are *n* = *m* = 5 objects/signals, meaning that the maximum possible payoff is *F*_*max*_ = 5. New agents sample their parent and *K* = 4 of their parent’s neighbors and record *k* = 1 observations for each object. Our testing suggests that these parameters do not affect the qualitative differences between the convergence dynamics of different network topologies, and mostly serve to affect the overall convergence time or level of noise in simulations. Neighbor influence is *δ* = 1, equivalent to neighbor-only learning. Networks have a similar average degree of 4. Results for a single configuration are averaged over 30 simulation runs each, except for results on adaptive networks, which are averaged over 16 runs each.

In order to examine convergence, or the level of communicability, in the population, we measure the average payoff of the population $F_{N}=\frac{1}{N}\sum_{I}^{N}F_{I}$, where *F*_*I*_ is the total payoff of agent *I* as defined in Eq 2. The final payoff *F*_*conv*_ is defined as *F*_*N*_ when the population has reached a stable or semi-stable state (which is usually, though not necessarily, the state where all agents have adopted a common language), or at the finite time step limit of the simulations *t*_*max*_. The convergence time *t*_*conv*_ is defined as the number of time steps before *F*_*N*_ reaches a value within some threshold *h* of the distance to *F*_*conv*_ (and stays within that threshold until *t*_*max*_ is reached). The relative distance between *F*_*N*_ and *F*_*conv*_ is defined as:

$$|\frac{F_{N}-F_{conv}}{F_{0}-F_{conv}}|,$$

where *F*_0_ is the initial payoff, or *F*_*N*_ at *t* = 0. In all simulations shown, *h* = 0.05.

**Fig 2:**
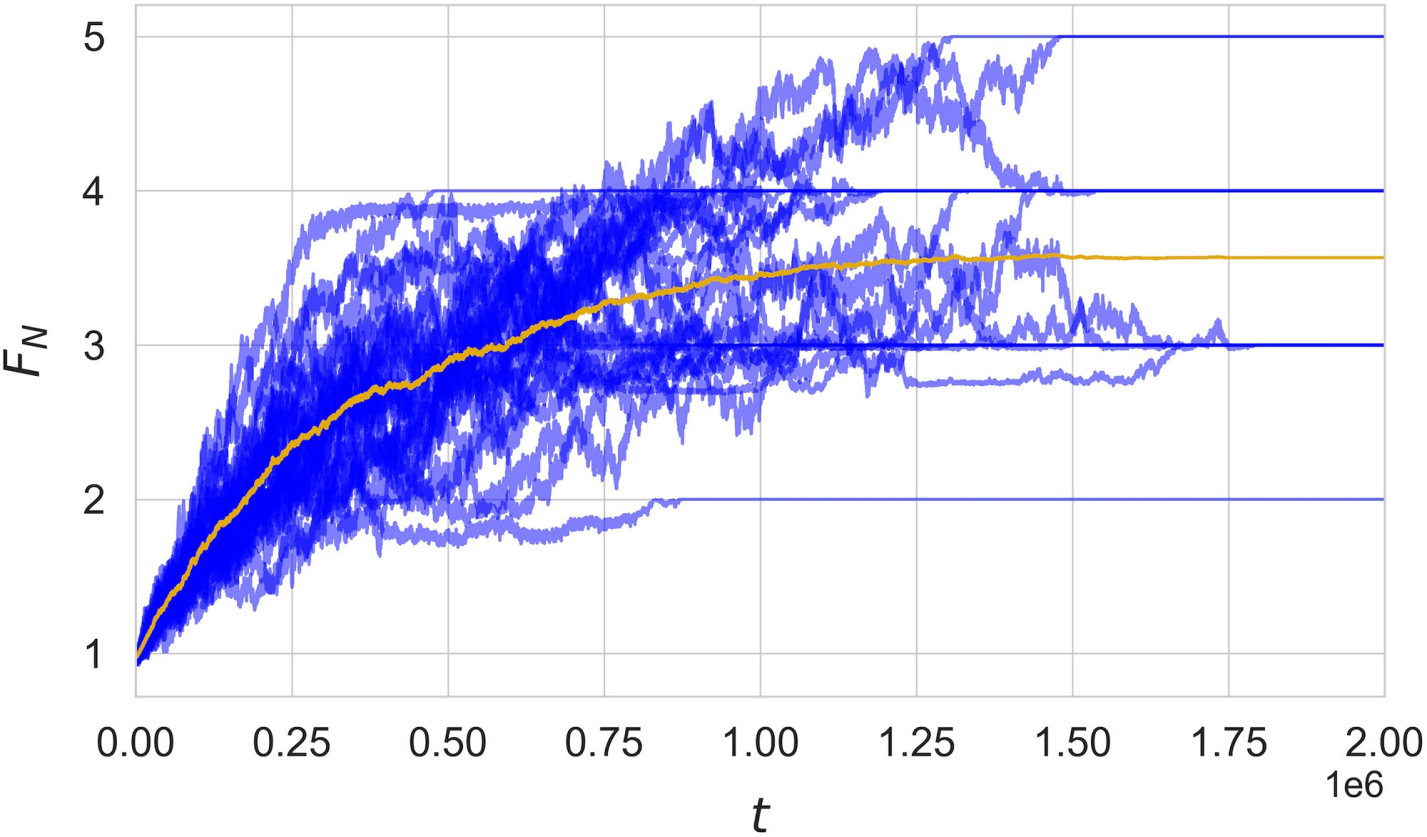
Example of evolutionary dynamics for various realizations of the Monte Carlo simulation and an averaged over 30 runs. The average payoff *F*_*N*_ is shown for both individual runs (blue lines) and their average (orange line). This example is for *N* = 400 run on a scale-free network, with *F*_*max*_ = 5 and *t*_*max*_ = 2 × 10^6^.

In general, starting from random languages in the initial generation, we see the population converge towards a shared language. In Fig 2, we plot the *F*_*N*_ over time for multiple simulation runs, as well as their average, in order to illustrate this convergence process. Average payoffs *F*_*N*_ fluctuate, generally increasing until they reach a quasi-stationary state, which typically happens when a single language described by binary permutation matrices is adopted by all agents. In this example, all runs eventually converge to a common language within *t*_*max*_, as indicated by static *F*_*N*_. However, this isn’t the case for all simulations, and sometimes payoffs can be observed to fluctuate indefinitely around some stable mean.

### Effects of population structure

**Fig 3:**
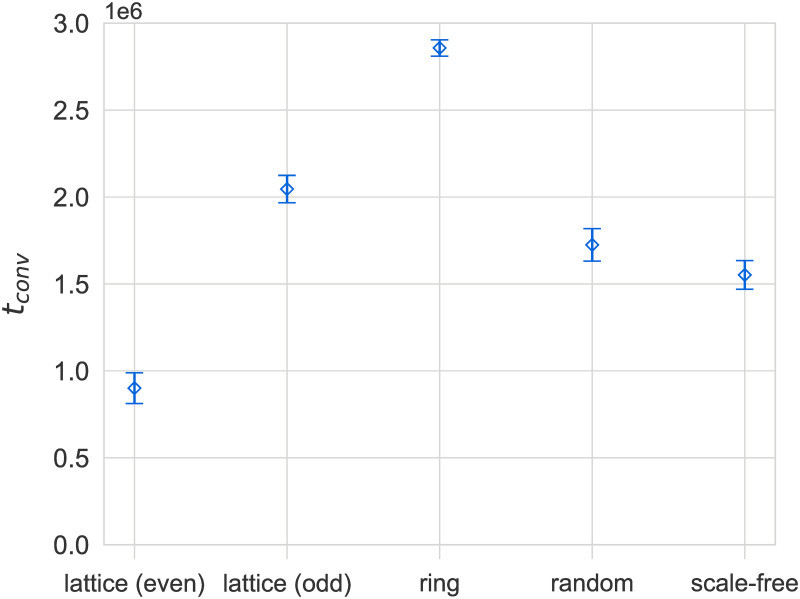
Differences in mean convergence time to a common language *t*_*conv*_ on different network topologies (left, plot) and average shortest path length *L* for all networks (right, table). Convergence times are roughly correlated with average shortest path lengths, with the exception of even-sized lattices. Results are for *N* = 500 and bars indicate standard errors.

**Fig 4:**
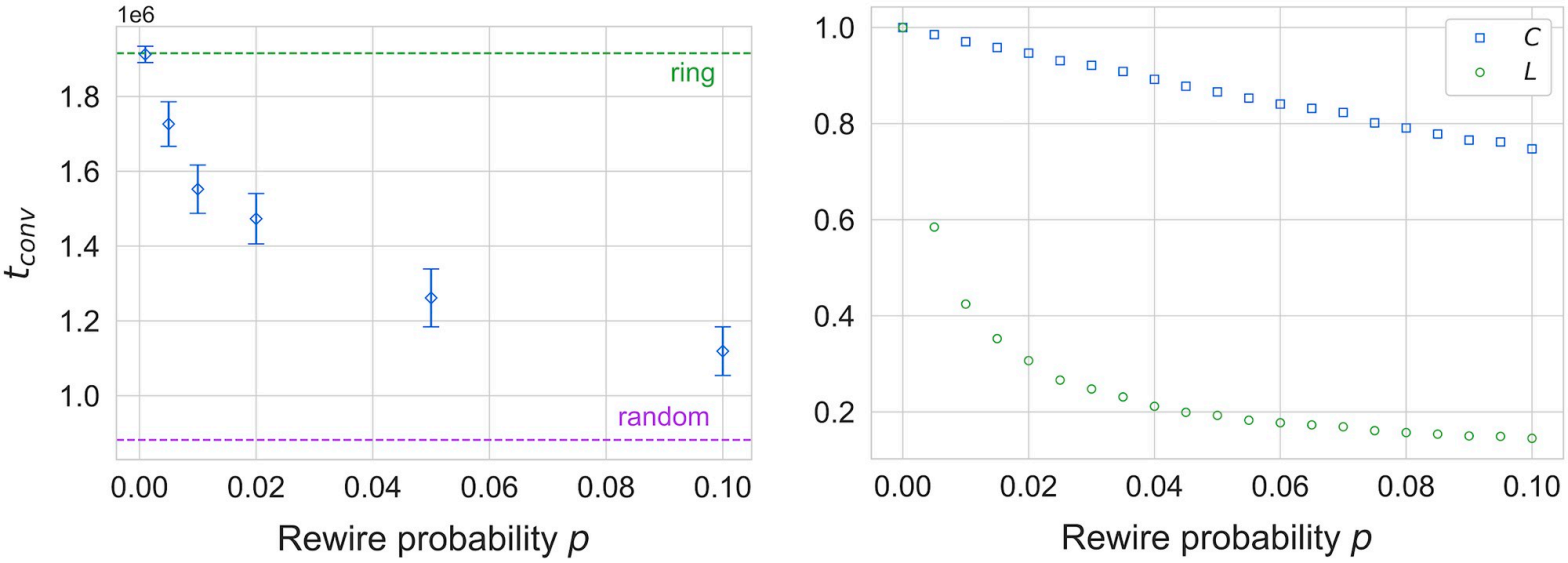
Scaling of convergence time *t*_*conv*_ (left) and network properties (right, average clustering *C* and average shortest path length *L*) for small-world networks, generated using the Watts-Strogatz model [44]. Average clustering *C* is defined as the average number of triangles, out of all possible triangles, that pass through a node, averaged over all nodes. Average shortest path length *L* is defined as the length of the shortest path connecting any two nodes on the network, averaged over all nodes. The convergence times of ring graphs and random networks are given, showing that small-world graphs approach the behavior of random networks as *p* increases, as expected. This is more likely a result of shorter average paths, which decrease sharply with an increase in *p*, while average clustering changes much more slowly for the same range. Results are for *N* = 400. Convergence times are averaged over 30 simulation runs each. Network properties are averaged over 50 network realizations.

**Fig 5:**
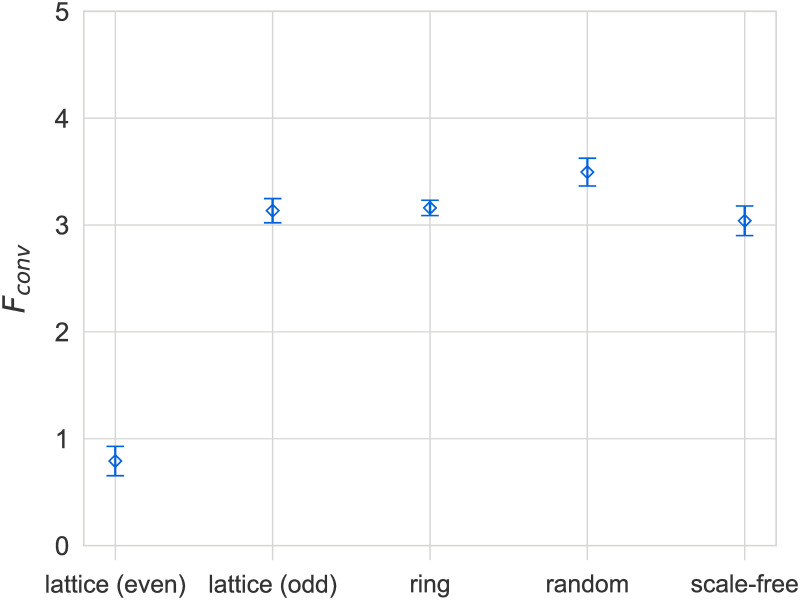
Differences in final payoffs of languages *F*_*conv*_ after convergence on different network topologies. There are no significant differences in final payoffs for different network topologies, except for even-sized lattices. Results are for *N* = 500 and bars indicate standard errors.

We begin by examining the effects of population structure on the convergence dynamics of the model for a population size of *N* = 500. As shown in Fig 3, we observe faster convergence on heterogeneous networks compared to spatially embedded topologies (2D lattices and rings), with the exception of even-sized lattices, which we discuss later. In particular, ring graphs exhibit noticeably slower convergence compared to other topologies, which could be attributed to one of two factors. First, the higher clustering on ring graphs could be slowing down convergence, since tightly coupled neighborhoods are more resistant to the propagation of new languages from the outside. Secondly, the shorter average path lengths on heterogeneous networks could be serving to accelerate the propagation of languages throughout the population. To investigate this effect further, we have also considered small-world networks built according to the Watts-Strogatz model [44]. By tuning the reconnection parameter *p*, in these networks, trade-offs between path-length and clustering can be investigated. Simulation results for the dependence of average convergence times *t*_*conv*_ on *p* are illustrated in Fig 4 (left). We also plot the dependence of the normalized clustering coefficient and average shortest path lengths for the same system size and range of *p* in Fig 4 (right). By comparing the dependencies in the left and right hand panel of Fig 4 we note the strong decline in convergence times in a regime of *p* in which average shortest path lengths decline strongly, whereas clustering remains essentially constant. This observation strongly suggests that *t*_*conv*_ is inversely related to path lengths. We also note that a similar effect has been observed for the NG [15]. It is worth noting that, in contrast to what has been observed in the NG [50], there is no difference between the convergence patterns of low- and high-degree nodes for our model (see data in S1 Fig). Further, as shown in Fig 5, the final payoff *F*_*conv*_ does not seem to be affected by the topology of the social network, again with the exception of even-sized lattices (discussed later).

#### Gridlocks on even-sized lattices

**Fig 6:**
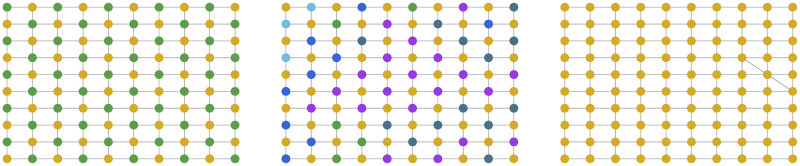
Demonstration of a gridlock pattern on 2D regular lattices. The pattern can either occur as two languages in a checkered pattern on the lattice (left), or as one dominant language distributed in a pattern, and multiple different languages in between (middle). Adding a single edge between any two nodes (right) disturbs the pattern and leads to a convergence similar to that of odd-sized lattices. A lattice with static boundaries is shown here for visualization purposes—periodic boundaries were used in simulations.

The differences in behavior between even-sized and odd-sized lattices owes to a convergence pattern on even-sized lattices that we have called gridlock—a stable state where at least one language emerges in a checkered spatial pattern, as shown in Fig 6. This pattern is very unstable, as it disappears when adding only a single edge between any two nodes on the graph (see the right hand panel of Fig 6). Gridlock also depends on specific learning parameters, namely the neighbor influence λ = 1 (i.e. learning purely from neighbors) and the sample size of *K* ≥ 4. Hence, it is likely a property of the specific topology and update rule of our configuration, and is not relevant to other configurations or real-world scenarios. As such, going forward we explore only odd-sized lattices. We nevertheless mention gridlocks because they are the only equilibrium state we have observed that can support multiple languages, although it does so at the expense of almost any communicability among the population. The failure to observe co-existence of languages in our model is notable because this phenomenon is important from a real-world perspective and it is often a feature of other models (see Ref. [51] for a review of language competition dynamics).

### Effects of population size

**Fig 7:**
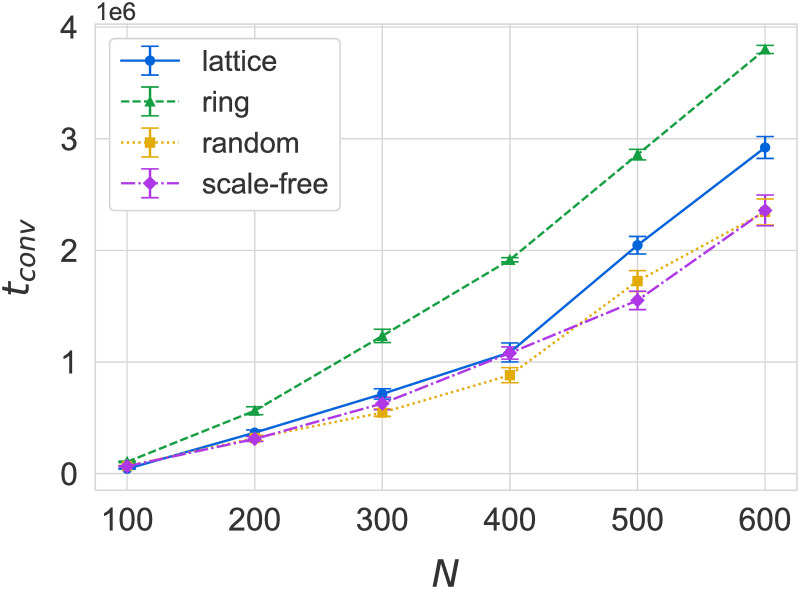
Scaling of convergence time *t*_*conv*_ with population size *N* on different networks. Sharper increases in *t*_*conv*_ correspond to larger average path lengths, although high clustering could also have an effect on ring graphs. Bars indicate standard errors.

Fig 7 shows the scaling of convergence time *t*_*conv*_ with population size *N*. We observe that final payoffs *F*_*conv*_ do not exhibit any notable change with *N* (see data in S2 Fig), so we have focused on the scaling of convergence time with system size. We find that *t*_*conv*_ for ring graphs and 2D lattices scale significantly worse compared to heterogeneous networks with an increase in population size, which could once again be attributed to the latter’s average shortest path length growing only logarithmically with *N*.

It is worthwhile to consider why final payoffs *F*_*conv*_ are not affected by population size or network topology. Natural language, in the view we have adopted, is an efficient and unambiguous system of mappings, but our results suggest that social structure is not a significant factor in enabling this. It has already been shown for similar models that linguistic factors, such as noise (from mistakes in learning [30]) and learning bias (towards one-to-one mappings [31]) can positively affect *F*_*conv*_ in the mean-field case (equivalent to a fully-connected network). However, despite the fact that degree heterogeneity introduces different levels of noise (from the composition of an agent’s neighborhood) and can significantly affect the fitness of agents (due to the local frequency-dependence of payoffs), our model does not show these topologies as having any effect on *F*_*conv*_. As already mentioned, computational constraints severely restrict explorable system sizes for our model on complex networks. Beyond what we have explored, we suspect that the effects of topology on some observables, such as the differences in final payoffs *F*_*conv*_, would be more pronounced for larger *N*. Another possibility is that the noise generated in the process of learning from neighbors drowns out the effects of topology. The language obtained by sampling neighbors during learning is an imperfect representation of the parent’s language. This limits the degree of reciprocity on the network compared to purely parental learning, which can weaken or blur different neighborhood effects that lead to convergence (fixation) in evolutionary games [52].

### Effects of neighbor influence

We move on to analyzing the effects of neighbor influence *δ* on the model’s dynamics. All of the results shown so far have been for *δ* = 1, in effect meaning that new agents sample their language only from their parent’s neighbors. This setting aims to simulate a process of purely cultural transmission of languages, and combined with the potential to sample from *K* = 4 neighbors, serves to introduce variance into the learning process. Of course, while language learning in the real world is heavily influenced by an individual’s broader social environment, it is clear that parents play a major role in this process, and it is unrealistic to exclude their influence entirely.

**Fig 8:**
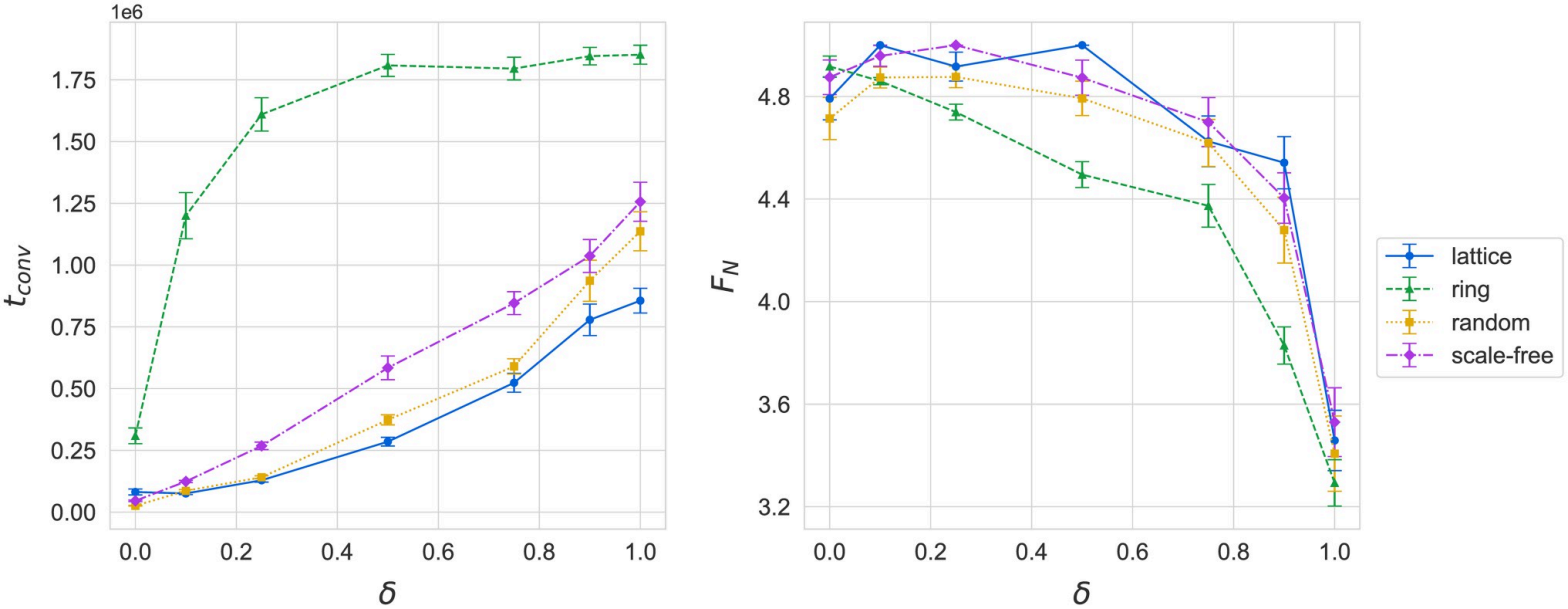
The effect of neighbor influence *δ* on the language dynamics of the model. Larger *δ* results in slower convergence and less optimal languages, except for the range *δ* ∈ (0.1, 0.2), where *F*_*conv*_ is maximized. Results are for *N* = 400, averaged over 24 runs each.

In general, as neighbor influence *δ* increases, the language of new agents moves from being a replication of its parent’s language, to an aggregate representation of languages in the neighborhood. Fig 8 shows simulation results for the dependence of convergence time *t*_*conv*_ and final payoffs *F*_*conv*_ on neighbor influence *δ*.

First, in Fig 8 (left), we see the dependence of convergence time *t*_*conv*_ on neighbor influence *δ*. We observe that a larger *δ* consistently leads to slower convergence, suggesting that reaching population-wide consensus is strictly faster when learning from parents rather than neighbors. Sampling an aggregate language from one’s neighbors (high *δ*), rather than mostly copying the parent’s language (low *δ*), introduces noise into the learning process and slows down adoption of existing effective languages. The magnitude of the change is perhaps more surprising, since a high payoff requires an agent that can effectively communicate with its neighbors, which in turn relies on the latter having languages that are roughly similar to the former’s. All network topologies exhibit very similar dependence on neighbor influence *δ*, with only ring graphs showing a noticeably slower convergence. The poor performance of ring topologies could be attributed to higher clustering, which makes it more difficult to “convert” tightly-knit neighborhoods with established languages that have a similar payoff to the dominant language.

Fig 8 (right) shows the dependence of final payoffs *F*_*conv*_ on neighbor influence *δ*. For heterogeneous (random and scale-free) networks, there is a range for the value of *δ* around (0.1, 0.2) that results in optimal languages. We suspect that within this range, sampling from neighbors introduces enough variance to improve the effectiveness of languages without completely obfuscating the effects of the selection pressure. This echoes the effect of noise in information transmission that has been recorded by other studies of evolutionary games. For large *δ*, learning is too noisy and drowns out the effects of selection for existing, high-payoff languages. This suggests that the result of averaging the languages of an agent’s neighbors is generally a poorer (more ambiguous) language than that of the parent, highlighting the role of vertical transmission for effective learning. More precise examination of the optimal range of neighbor influence *δ* in terms of *F*_*conv*_ is left for a future study.

We suspect the effects of learning influence would be heavily affected by the strength of the selection pressure towards agents with a higher payoff. For small neighbor influence *δ*, there is very little adaptation, in the form of “innovations” introduced at the learning stage, and the dynamics mostly rely on efficient languages already existing in the initial generation. As we have seen, even a small amount of influence beyond *δ* = 0 can nudge the dynamics towards a more optimal state. However, a stronger selection pressure would amplify how quickly existing languages propagate and increase the likelihood of the dynamics settling into a sub-optimal equilibrium. This would shift the optimal range for *δ* in respect to *F*_*conv*_, potentially requiring a larger *δ* to balance out.

### Effects of adaptive rewiring

**Fig 9:**
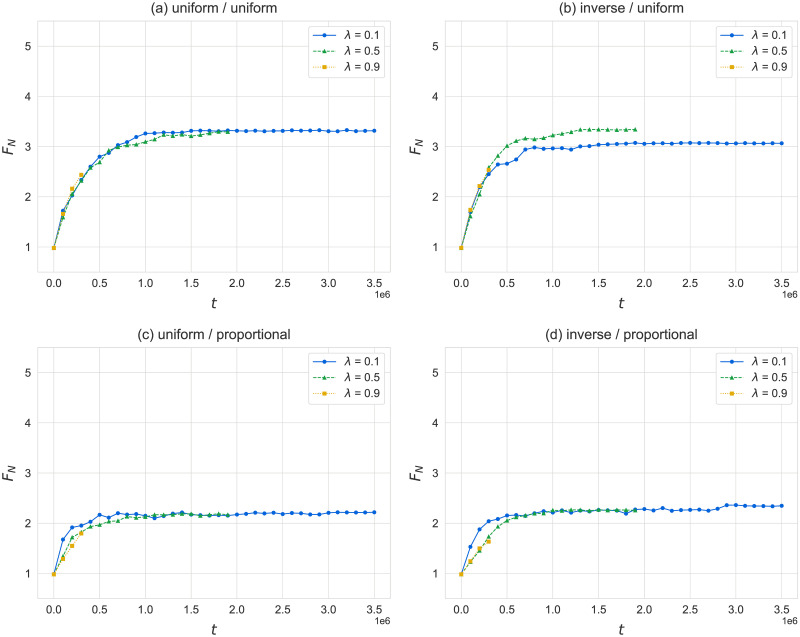
Effects of different rewiring rules and rewire probability λ on average payoffs *F*_*N*_. We show (a) uniform disconnection and uniform reconnection, as a base case; (b) fitness-inverse disconnection and uniform reconnection; (c) uniform disconnection and fitness-proportional reconnection; and (d) fitness-inverse disconnection and fitness-proportional reconnection (see previous section for definition of these rewiring rules.) Three different values of λ are shown for each configuration of rewire rules: 0.1, 0.5, and 0.9. The x-axis is normalized for the number of reproduction events. Simulation complexity and limits on computational resources did not allow us to simulate all cases for an equal number of reproduction steps. Results are for random networks and *N* = 400, with neighbor influence *δ* = 0.5, averaged over 16 runs.

**Fig 10:**
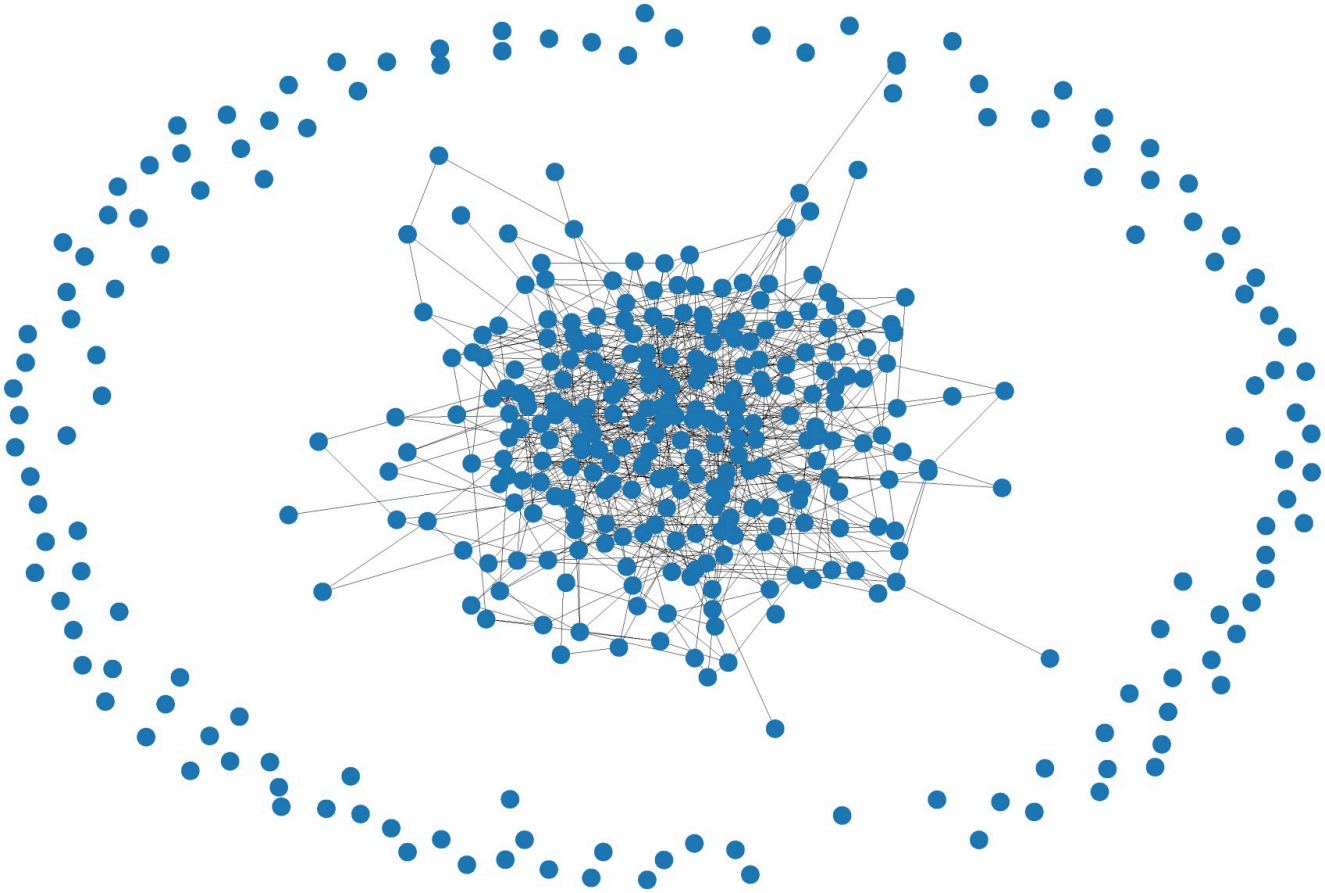
Illustration of core-periphery structure as a result of reconnecting with fitness-proportional probability. The central cluster has a degree distribution identical to that of a random network.

In this section, we investigate the effects of a co-evolution of state and topology through adaptive rewiring rules, as defined in the previous section. Fig 9 shows the dependence of average payoffs *F*_*N*_ on the choice of rewiring rule and the rewire probability λ on random networks for a population size *N* = 400. We have also included a comparison of the degree distributions at the start and end of simulations in S3 Fig. For uniform reconnection (panels (a) and (b)) both the convergence pattern and the payoffs resemble what we see on random networks with no rewiring (λ = 0). The resulting networks also have the same degree distribution as random networks (refer to data in S3 Fig). For fitness-proportional reconnection (panels (c) and (d)) we see lower payoffs and marginally faster convergence compared to uniform reconnection. As illustrated in Fig 10, in these cases the evolution of networks results in a core-periphery structure, with an average of almost 25% of agents completely isolated and the remaining agents forming a dense cluster that exhibits properties of a random network. This occurs despite the addition of a 10% chance to reconnect to a random agent in the population, and results in lower payoffs for the population overall, since isolated agents are considered as having a payoff of 0. Agents in the dense, central cluster achieve the same payoffs as for uniform reconnection or non-adaptive random networks.

When comparing panels (a) vs. (c) and (b) vs. (d) in Fig 9, we can see that disconnecting with a fitness-inverse probability does not have a significant effect on convergence. A possible impact can be observed in panel (b) for uniform reconnection, where fitness-inverse disconnection results in lower average payoffs *F*_*N*_ when the rewire probability λ is low, but the noise inherent in the results makes it difficult to conclude whether this is a true effect. Indeed, we once again suspect that the lack of any significant impact of adaptive rewiring on the dynamics of the model is due to the noise introduced by learning from neighbors. For purely parental learning, when the effects of reciprocity are stronger, disconnection would be more targeted towards poorly-communicating agents, which could speed up convergence. Additionally, an interesting dynamic might form if agents had an easier time “converting” their neighbors, as in purely parental learning, by replacing them with a child that has a near-exact copy of their language. In that case, agents with fewer neighbors would achieve higher payoffs faster, and would be favored for reconnection, which would in turn decrease their payoffs as they would likely not be good at communicating with their new connections.

## Conclusion

In this study, we have presented a model for the emergence of language as shared object-meaning associations through evolutionary dynamics within a structured population of agents. We have explored the change in speed of convergence and level of communicability between agents stemming from the interplay between topological and linguistic factors. We find that small-world effects stemming from short average path lengths contribute to faster convergence. We also see a significant impact of the type of transmission, specifically in comparison between vertical vs. oblique transmission, on both convergence time and the final payoffs of languages. We also observe that the model’s dynamics under adaptive rewiring of the underlying network can result in isolation of nodes when agents preferentially rewire to other well-communicating members of the population. Lastly, we note that a number of factors do not seem to influence the dynamics of language formation, namely network heterogeneity and some forms of adaptive rewiring. We suspect this has to do with the effects of learning from neighbors, which introduces noise into the learning/transmission process and limits the effects of reciprocity, thus obscuring the impact of topology.

The complexity and specificity of our model raises the question whether any of our results can be said to contain universal findings on the dynamics of language evolution. The difficulty in showcasing universality of experimental findings lies in the large number of possible modelling choices. In the current study, we have taken care to explore as many aspects of the model configuration as possible (also see additional simulation results in S4 and S5 Figs), focusing on those that have been established as significant in previous work (e.g. small-world effects [53, 54], learning dynamics [12], adaptive rewiring [55]). The complexity of our model stems mainly from the attempt to consider both the effects of network topology and reproduction/learning dynamics simultaneously. Unavoidably, this complexity limits the universality of our findings. However, we believe that having focused on modelling choices common in the related literature, our study provides important comparisons to other models (like we have done with the NG) and informs future work concerning the impact of topology in particular, which has been understudied for evolutionary models in the field.

In light of the discussion on the model’s complexity, we believe there is ample room for future study. Most importantly, our results suggest that it would be worthwhile to explore the questions we have tried to answer here in the context of the parental-neighbor (vertical-oblique) learning trade-off. Specifically, a smaller influence of neighbors would make the dynamics easier to interpret and we suspect would allow us to isolate different topological effects more easily. Additionally, there are a number of parameters that we have not explored in this study. Topological properties of different complex networks, such as average degree, clustering, community structure, and assortative mixing have been shown to affect dynamics in other models. Evolutionary and learning parameters, such as the sample size *K*, the choice of update rule, and the strength of selection have also not been explored, but we suspect could yield interesting results. Finally, it would be beneficial for the robustness of these and future results to address the resource availability and demand constraints of simulations, which would allow for the exploration of larger system sizes and more accurate estimations of mean values.

We started by comparing the ELG, which is the original inspiration for our model, to the popular NG model due to the availability of data on the effects of social structure on the model’s dynamics. As we have mentioned on a few occasions in the previous section, the effects of topology on our model seem to differ from those observed for the NG. It would be worth investigating further why this is the case, and whether the dynamics of our model can be brought to a similar time scale as those of the NG. In general, carrying out cross-model comparisons between models that examine different linguistic factors and/or proceed on different time scales might be the only way to draw robust conclusions about the effects of social structure on language evolution [6]. The complexity of models themselves, and the difficulty in carrying out such comparisons, only emphasizes the importance of employing computational tools in this field.

## Supporting information

S1 Fig Differences in convergence patterns for low- and high-degree nodes. Average payoffs *F*_*N*_ are shown for the top 10% and bottom 10% of nodes, ranked by their degree on the network. No differences in the pattern of convergence and the magnitude of payoffs can be observed. Results are for scale-free network and *N* = 500, with neighbor influence *δ* = 1 and no rewiring.
(TIF)

S2 Fig Scaling of final payoffs *F*_*conv*_ with population size *N* on different networks. No significant difference in payoffs can be observed. See main text for discussion on why this might be the case.
(TIF)

S3 Fig Comparison of degree distributions of networks using adaptive rewiring at the start and end of simulations for different rewire probability λ. Degree distributions of the network at the start (*t* = 0) are shown in blue and distributions at the end (*t* = *t*_*max*_) are shown in orange. Rows correspond to different setups with respect to rewiring rules and columns correspond to different rewiring probabilities λ. The second and fourth rows show the case of disconnecting with fitness-inverse probability. If we compare them to the case of uniform disconnection (rows one and three), we see that fitness-inverse disconnection does not affect the change in degree distributions. The top two rows show reconnection with uniform probability, while the bottom two show reconnection with fitness-proportional probability. We can see that fitness-proportional disconnection results in a large number of nodes isolated from the rest of the population (equivalent to a degree *d* = 0). The rest of the nodes form a cluster whose degree distribution resembles that of a random network. These central clusters have marginally higher average and maximum degree compared to the networks at the start of the simulation. If we compare degree distributions along columns, we do not see any differences caused by the rewire probability λ. All simulations shown started with random networks. Results are for *N* = 400 and neighbor influence *δ* = 1.
(TIF)

S4 Fig Evolution of average payoffs *F*_*N*_ over time on fully-connected networks subject to different neighbor influence *δ*. For *δ* = 0, equivalent to purely parental learning, convergence is fast and almost optimal (considering maximum payoffs of 5). For *δ* = 1, equivalent to purely neighbor learning, convergence is much slower and languages have lower average payoffs *F*_*N*_. For *δ* = 0.5, there is a balance between the convergence speed and payoff of languages, where *F*_*conv*_ is maximized. These results are consistent with what we have observed for other network structures, as shown in Fig 8, suggesting that the noise introduced by neighbor influence *δ* impairs convergence, but that in smaller amounts it can promote it instead. Results are for *N* = 200, instead of 400, due to the larger computational demands of simulations on fully-connected networks.
(TIF)

S5 Fig Evolution of average payoffs *F*_*N*_ over time on random regular graphs subject to different neighbor influence *δ*. Results are similar to those presented for other network structures, as discussed for Fig 8 and S4 Fig. Briefly, for *δ* = 0 convergence is fast, while for *δ* = 1 it is much slower and languages have lower average payoffs *F*_*N*_. For *δ* = 0.5, we see a balance, whereby *F*_*conv*_ is maximized while convergence remains relatively fast. Results are for *N* = 400 on random 4-regular graphs.
(TIF)

S1 TableLanguage competition and the scale-merit effect in language evolution.An important phenomenon observed in the evolution of languages in the real world is the scale-merit or bandwagon effect, whereby more popular languages are preferred by speakers for their higher utility, and thereby become even more widespread in the population. As discussed previously, our model includes a bias towards more popular languages, since the payoffs of any individual agent will depend on the frequency of languages represented in its immediate neighbourhood. To showcase this effect, we have conducted a series of simulations of language competition, which proceed as follows. Instead of initializing the population with random languages, we generate two languages *A* and *B*, that yield the same payoffs with respect to themselves. These languages are then distributed randomly among the population of agents in given proportions (see first row of table). Reproduction and learning dynamics proceed as normal. The results shown in the table are for random networks, *N* = 400, *δ* = 1, and λ = 0. We observe that the proportion of simulation runs that result in *A* being dominant, i.e. the population reaches a stable state where all agents speak *A*, increases with the number of *A* agents in the initial population. Having no other advantage over *B*, this shows that more numerous languages tend to be more successful in the final population. Additionally, we have shown the average similarity for languages in the population both at the start and end of the simulations. Similarity of a language *C* to language *A* is defined as $1-\frac{H(A,C)}{n\times m}$, where *H* is the Hamming distance between the two languages, and *m* × *n* is the maximum Hamming distance given *n* objects and *m* signals. The average similarity is calculated using a weighted arithmetic mean over the distribution of languages in the population. We see that similarities remain stable, with already popular languages maintaining popularity or giving rise to similar languages by the end of the simulations. There is no drastic convergence to either of the initial languages, primarily due to the averaging effects and noise introduced by neighbor sampling (*δ* = 1), which slows down and softens convergence (see main text for further discussion on this point).(TIF)
