# Supplementary figures and images for: On the evolutionary language game in structured and adaptive populations
Source: PLoS One. 2022 Aug 30;17(8):e0273608. doi: 10.1371/journal.pone.0273608 (PMC9426894; doi:10.1371/journal.pone.0273608)

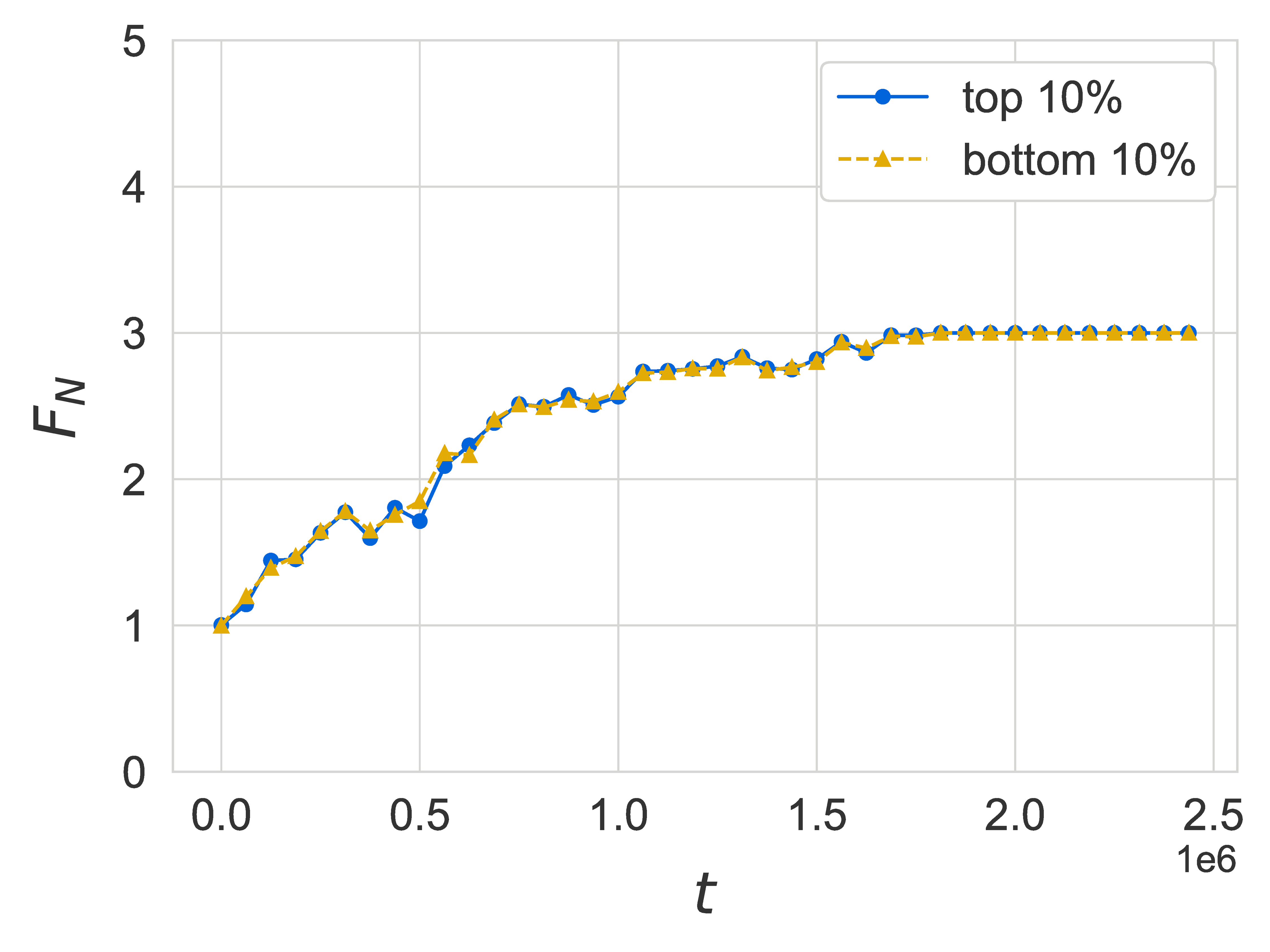

Supplement: S1 Fig — Average payoffs FN are shown for the top 10% and bottom 10% of nodes, ranked by their degree on the network. No differences in the pattern of convergence and the magnitude of payoffs can be observed. Results are for scale-free network and N = 500, with neighbor influence δ = 1 and no rewiring. (TIF) [file pone.0273608.s001.tif]

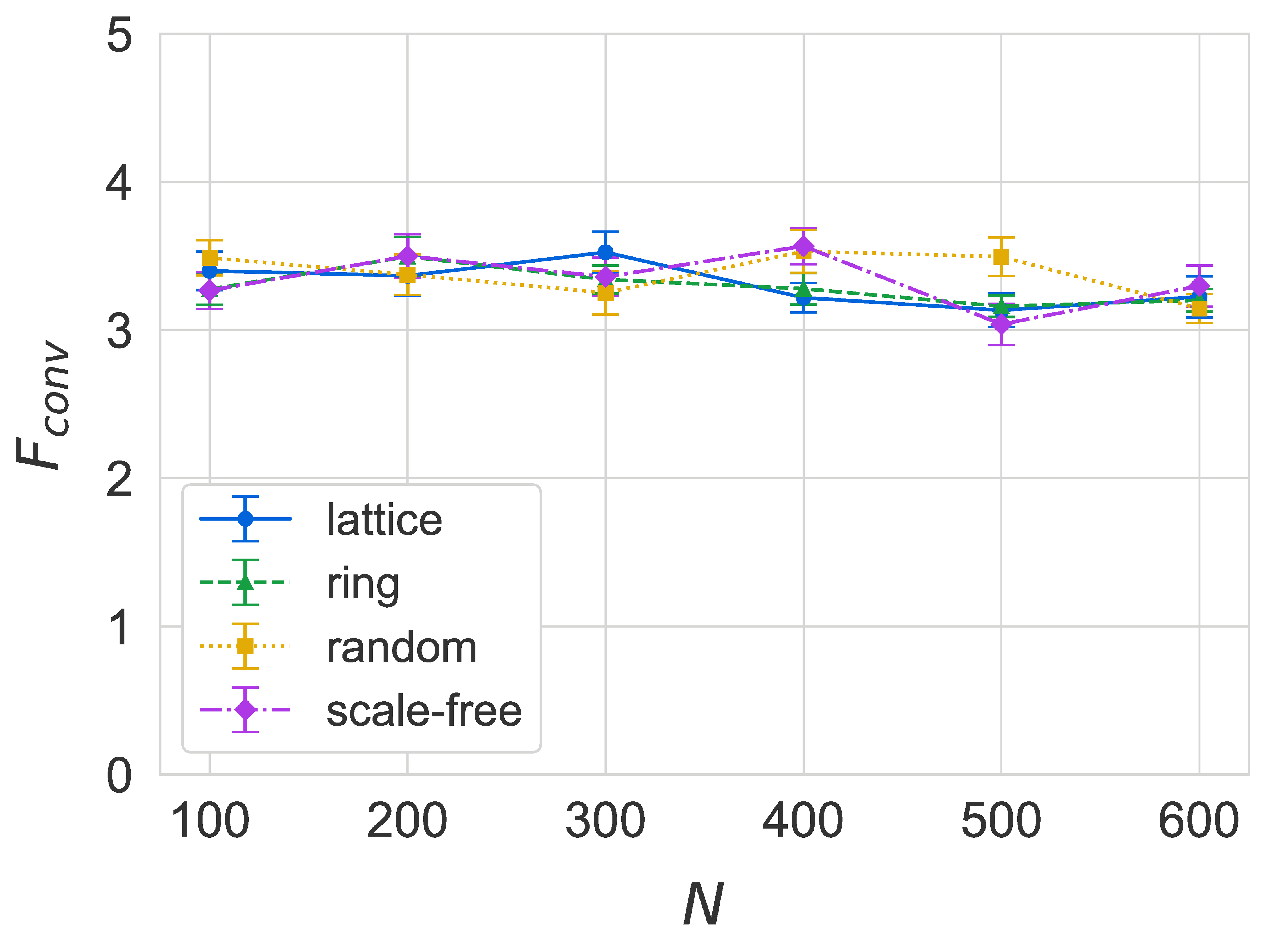

Supplement: S2 Fig — No significant difference in payoffs can be observed. See main text for discussion on why this might be the case. (TIF) [file pone.0273608.s002.tif]

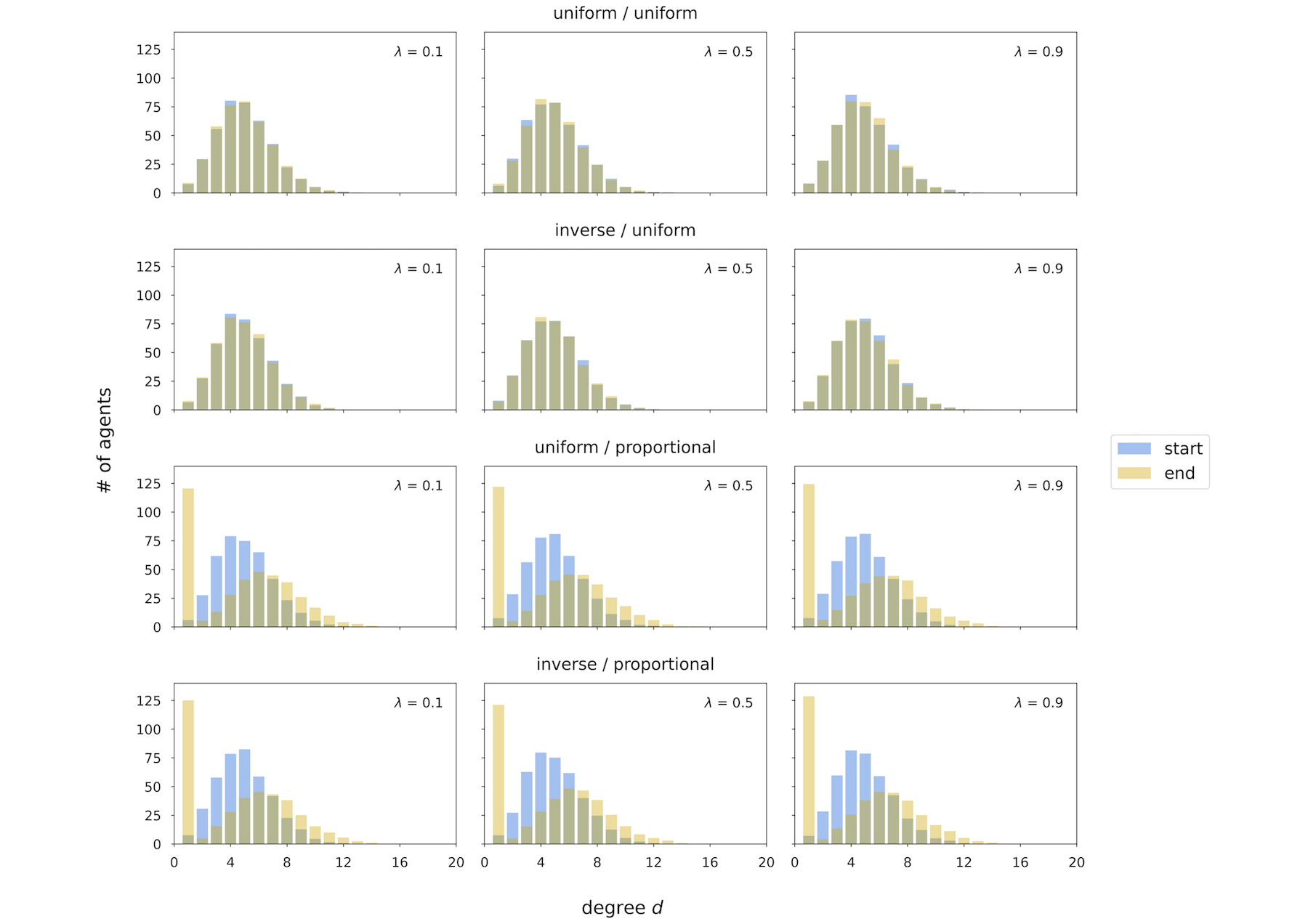

Supplement: S3 Fig — Degree distributions of the network at the start (t = 0) are shown in blue and distributions at the end (t = tmax) are shown in orange. Rows correspond to different setups with respect to rewiring rules and columns correspond to different rewiring probabilities λ. The second and fourth rows show the case of disconnecting with fitness-inverse probability. If we compare them to the case of uniform disconnection (rows one and three), we see that fitness-inverse disconnection does not affect the change in degree distributions. The top two rows show reconnection with uniform probability, while the bottom two show reconnection with fitness-proportional probability. We can see that fitness-proportional disconnection results in a large number of nodes isolated from the rest of the population (equivalent to a degree d = 0). The rest of the nodes form a cluster whose degree distribution resembles that of a random network. These central clusters have marginally higher average and maximum degree compared to the networks at the start of the simulation. If we compare degree distributions along columns, we do not see any differences caused by the rewire probability λ. All simulations shown started with random networks. Results are for N = 400 and neighbor influence δ = 1. (TIF) [file pone.0273608.s003.tif]

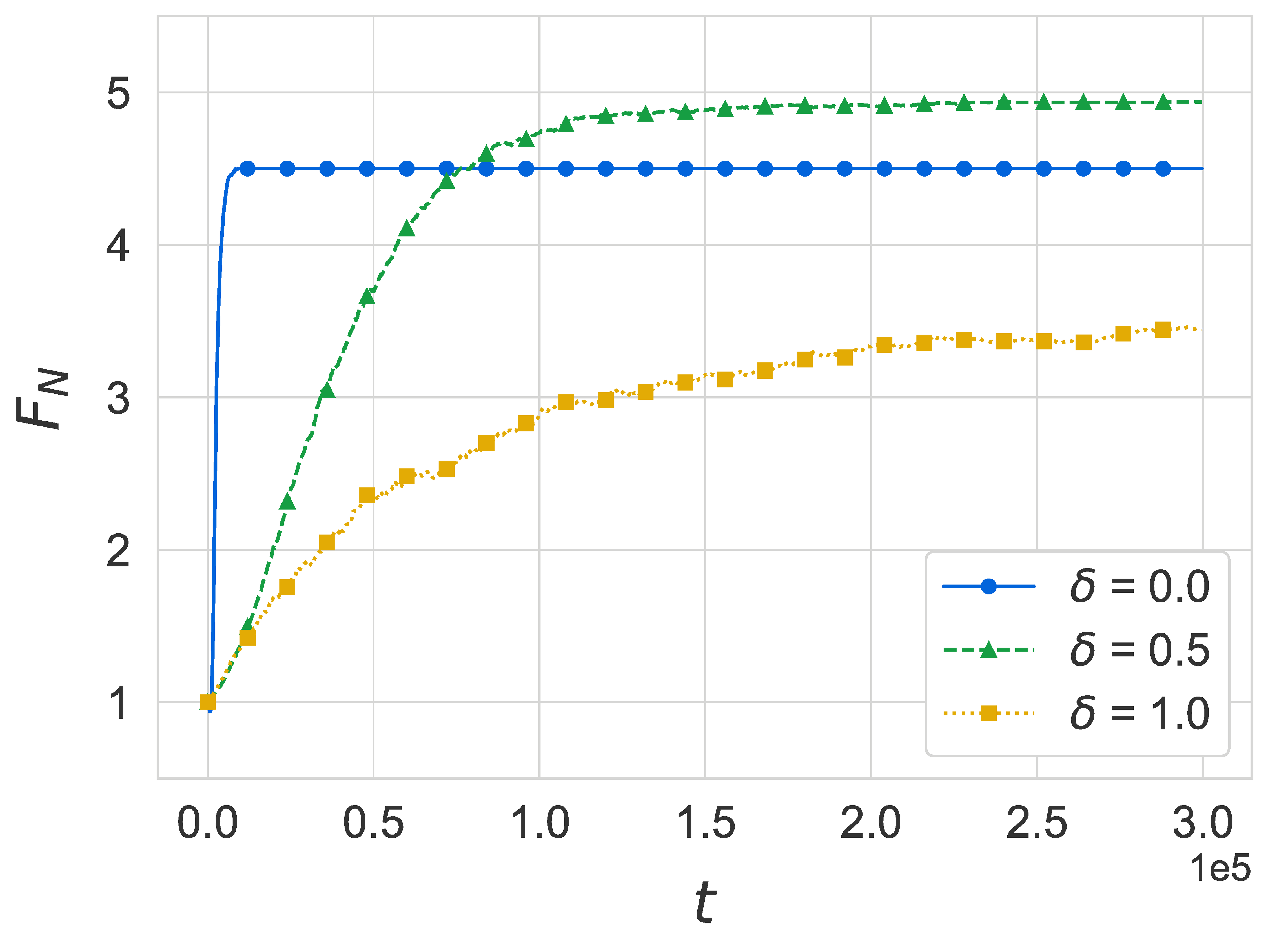

Supplement: S4 Fig — For δ = 0, equivalent to purely parental learning, convergence is fast and almost optimal (considering maximum payoffs of 5). For δ = 1, equivalent to purely neighbor learning, convergence is much slower and languages have lower average payoffs FN. For δ = 0.5, there is a balance between the convergence speed and payoff of languages, where Fconv is maximized. These results are consistent with what we have observed for other network structures, as shown in Fig 8, suggesting that the noise introduced by neighbor influence δ impairs convergence, but that in smaller amounts it can promote it instead. Results are for N = 200, instead of 400, due to the larger computational demands of simulations on fully-connected networks. (TIF) [file pone.0273608.s004.tif]

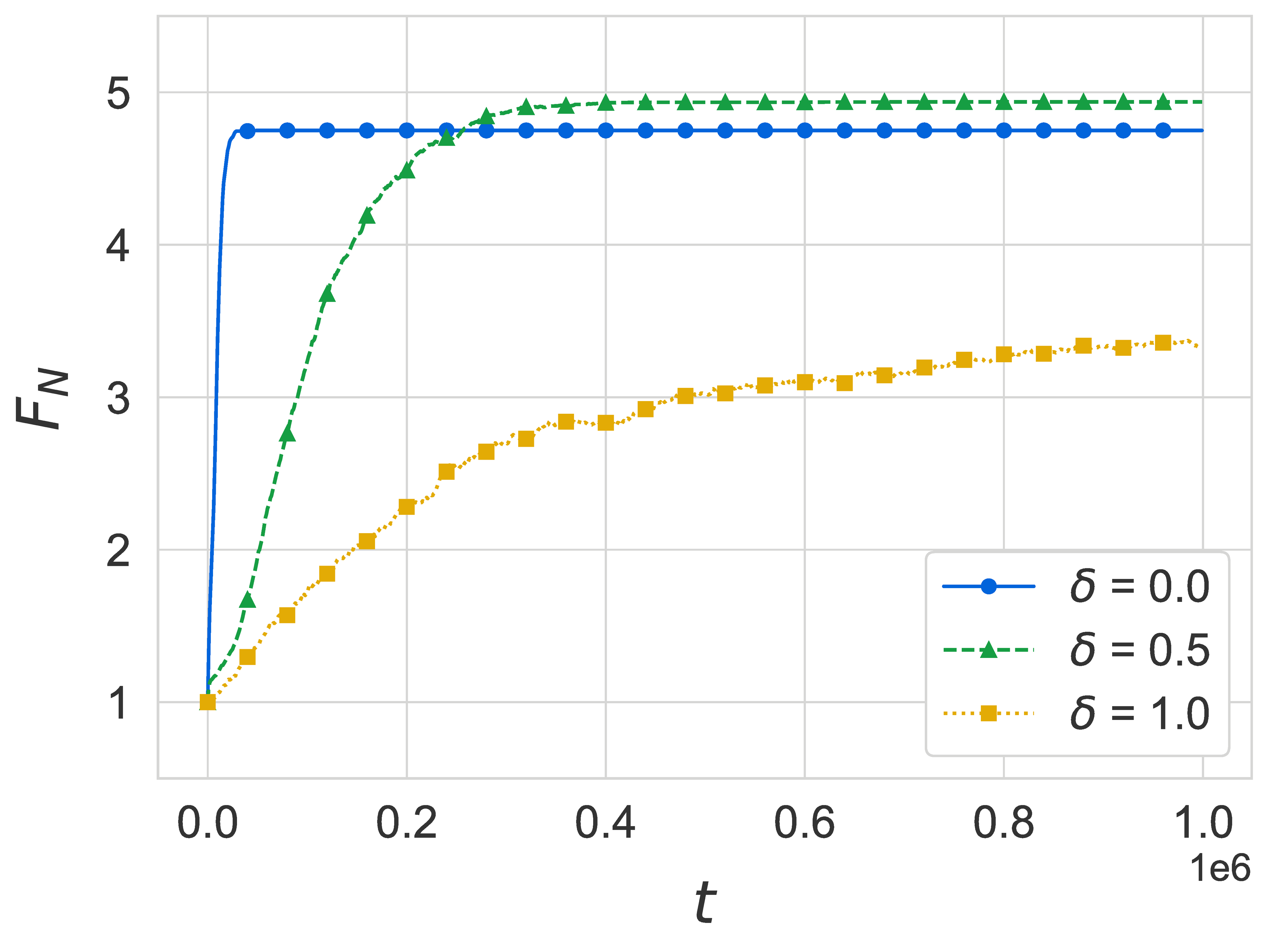

Supplement: S5 Fig — Results are similar to those presented for other network structures, as discussed for Fig 8 and S4 Fig. Briefly, for δ = 0 convergence is fast, while for δ = 1 it is much slower and languages have lower average payoffs FN. For δ = 0.5, we see a balance, whereby Fconv is maximized while convergence remains relatively fast. Results are for N = 400 on random 4-regular graphs. (TIF) [file pone.0273608.s005.tif]

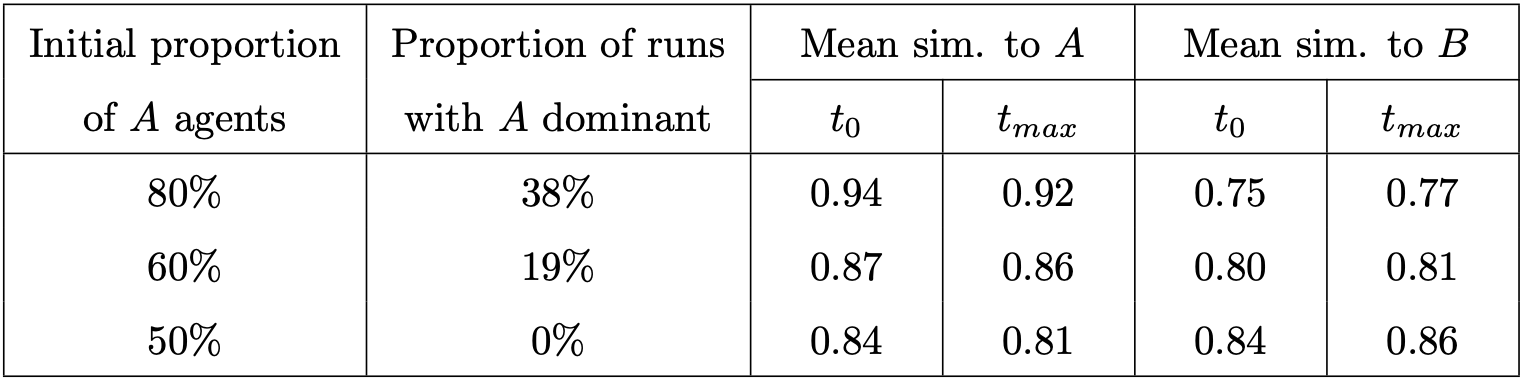

Supplement: S1 Table — An important phenomenon observed in the evolution of languages in the real world is the scale-merit or bandwagon effect, whereby more popular languages are preferred by speakers for their higher utility, and thereby become even more widespread in the population. As discussed previously, our model includes a bias towards more popular languages, since the payoffs of any individual agent will depend on the frequency of languages represented in its immediate neighbourhood. To showcase this effect, we have conducted a series of simulations of language competition, which proceed as follows. Instead of initializing the population with random languages, we generate two languages A and B, that yield the same payoffs with respect to themselves. These languages are then distributed randomly among the population of agents in given proportions (see first row of table). Reproduction and learning dynamics proceed as normal. The results shown in the table are for random networks, N = 400, δ = 1, and λ = 0. We observe that the proportion of simulation runs that result in A being dominant, i.e. the population reaches a stable state where all agents speak A, increases with the number of A agents in the initial population. Having no other advantage over B, this shows that more numerous languages tend to be more successful in the final population. Additionally, we have shown the average similarity for languages in the population both at the start and end of the simulations. Similarity of a language C to language A is defined as 1-H(A,C)n×m, where H is the Hamming distance between the two languages, and m × n is the maximum Hamming distance given n objects and m signals. The average similarity is calculated using a weighted arithmetic mean over the distribution of languages in the population. We see that similarities remain stable, with already popular languages maintaining popularity or giving rise to similar languages by the end of the simulations. There is no drastic convergence to ei [file pone.0273608.s006.tif]
